# Post-Reconstitution Hemostatic Stability Profiles of Canadian and German Freeze-Dried Plasma

**DOI:** 10.3390/life14020172

**Published:** 2024-01-24

**Authors:** Henry T. Peng, Katherine Moes, Kanwal Singh, Shawn G. Rhind, Chantale Pambrun, Craig Jenkins, Luis da Luz, Andrew Beckett

**Affiliations:** 1 Defence Research and Development Canada, Toronto Research Centre, Toronto, ON M3K 2C9, Canada; 2 St. Michael’s Hospital, University of Toronto, Toronto, ON M5B 1W8, Canada; kanwal.singh@unityhealth.to (K.S.); andrew.beckett@unityhealth.to (A.B.); 3 Centre for Innovation, Canadian Blood Services, Ottawa, ON K1G 4J5, Canada; chantale.pambrun@blood.ca (C.P.); craig.jenkins@blood.ca (C.J.); 4 Sunnybrook Health Sciences Centre, University of Toronto, Toronto, ON M4N 3M5, Canada; luis.daluz@sunnybrook.ca; 5 Royal Canadian Medical Services, Ottawa, ON K1A 0K2, Canada

**Keywords:** freeze-dried plasma, coagulation, stability, hemostatic resuscitation, prehospital transfusion

## Abstract

Despite the importance of the hemostatic properties of reconstituted freeze-dried plasma (FDP) for trauma resuscitation, few studies have been conducted to determine its post-reconstitution hemostatic stability. This study aimed to assess the short- (≤24 h) and long-term (≥168 h) hemostatic stabilities of Canadian and German freeze-dried plasma (CFDP and LyoPlas) after reconstitution and storage under different conditions. Post-reconstitution hemostatic profiles were determined using rotational thromboelastometry (ROTEM) and a Stago analyzer, as both are widely used as standard methods for assessing the quality of plasma. When compared to the initial reconstituted CFDP, there were no changes in ROTEM measurements for INTEM maximum clot firmness (MCF), EXTEM clotting time (CT) and MCF, and Stago measurements for prothrombin time (PT), partial thromboplastin time (PTT), D-dimer concentration, plasminogen, and protein C activities after storage at 4 °C for 24 h and room temperature (RT) (22–25 °C) for 4 h. However, an increase in INTEM CT and decreases in fibrinogen concentration, factors V and VIII, and protein S activities were observed after storage at 4 °C for 24 h, while an increase in factor V and decreases in antithrombin and protein S activities were seen after storage at RT for 4 h. Evaluation of the long-term stability of reconstituted LyoPlas showed decreased stability in both global and specific hemostatic profiles with increasing storage temperatures, particularly at 35 °C, where progressive changes in CT and MCF, PT, PTT, fibrinogen concentration, factor V, antithrombin, protein C, and protein S activities were seen even after storage for 4 h. We confirmed the short-term stability of CFDP in global hemostatic properties after reconstitution and storage at RT, consistent with the shelf life of reconstituted LyoPlas. The long-term stability analyses suggest that the post-reconstitution hemostatic stability of FDP products would decrease over time with increasing storage temperature, with a significant loss of hemostatic functions at 35 °C compared to 22 °C or below. Therefore, the shelf life of reconstituted FDP should be recommended according to the storage temperature.

## 1. Introduction

Massive hemorrhage remains the main cause of preventable death on the battlefield [1] and the leading cause of death worldwide in people aged 18 to 39 years, with nearly half occurring in the prehospital setting [2,3]. Damage Control Resuscitation (DCR) with blood products is recommended at the earliest possible time after combat injuries and major trauma [3,4]. However, there is no consensus on an optimal transfusion strategy with various options for blood products [5,6].

Liquid and dried blood products are available for DCR. The former includes whole blood, fresh frozen plasma, red blood cells, platelet concentrate, and cryoprecipitate; the latter includes freeze-dried plasma (FDP) and clotting factor concentrates (e.g., fibrinogen, prothrombin complex). Current evidence does not show a definitive survival benefit of FDP compared to crystalloids in prehospital hemorrhage management [7,8,9]; however, FDP may be beneficial in cases of severe hemorrhagic shock, prolonged transport times, and in the absence of tranexamic acid. Dried blood products have logistic advantages over their liquid counterparts for transfusion in far-forward military settings and massive casualties’ events since they are temperature stable, lightweight, safe, do not require refrigeration and thawing processes, and thus can be easily carried and rapidly administered in an austere field environment [10].

Currently, when stored at −20 °C, fresh frozen plasma (FFP) has an expected shelf life of two years [11]. FFP must be thawed prior to use. The thawing process takes approximately 30 min. After thawing, FFP must be transfused immediately or refrigerated and used within five days [12]. This presents significant logistical challenges, resulting in a considerable delay in receiving plasma.

Given the aforementioned logistic advantages, dried plasma is a promising alternative to FFP for hemostatic resuscitation at a large quantity and relatively low cost in prehospital settings, such as on the battlefield, at sea, and during aeromedical evacuation [13]. The product contains a full spectrum of coagulation factors, which may be required in severe bleeding trauma patients. The use of lyophilized plasma also allowed rapid and high-ratio transfusion practices in severe trauma patients, which could increase survival [14,15].

Because of the benefits of early plasma resuscitation and the current state of technology, dried plasma may be the best option for blood products when and where needed [16]. The need for dried plasma to support casualties in far-forward austere environments will increase in future multi-domain operations as a result of high casualty projections and prolonged field care paradigms, as well as significantly more challenging logistic considerations and limitations of blood products for massive transfusion [16,17]. Additionally, the COVID-19 pandemic and possible large conflicts in the foreseeable future highlight the need to stockpile blood products with long shelf lives (e.g., dried plasma) to be sufficient for medical care in an emergency [18] and for massive combat casualties [19].

Currently, there are three licensed dried plasma products, which are all FDP from different sources [13]. The French Army’s Centre Transfusion Sanguine des Armees has developed FLYP using pooled apheresis plasma from 10 carefully screened and monitored donors, while the German Red Cross has developed LyoPlas from single-donor plasma, quarantine-stored for at least 4 months. The plasma is frozen below −30 °C in a separate step, followed by lyophilization in specially designed freeze-dryers for several days to reduce water content below 1% [20]. In Europe, both FLYP and LyoPlas have been in limited use in military and civilian prehospital settings. The National Bioproducts Institute of South Africa has produced a pooled, solvent/detergent, ABO-universal, lyophilized plasma called Bioplasma FDP from hundreds of donors (up to 1500). These products generally have the same indications for use as other forms of plasma or pathogen-reduced plasma [21]. Published reports have documented more than 600 patients who have received FLYP or LyoPlas in prehospital and austere environments, with the majority receiving LyoPlas [10].

However, these products do not meet regulatory requirements by Health Canada concerning the risk of prion disease transmission caused by misfolded proteins. In addition, these products are produced in a glass bottle that is not ruggedized for combat environments. There is an opportunity to produce new FDP from a large amount of available AB Rh Pos universal donor plasma, which otherwise may be inefficiently utilized in Canadian hospitals due to product expiration. In particular, our Canadian FDP (CFDP) is produced from Canadian-sourced plasma using the Terumo BCT freeze-drying system in a rugged, light-weight plastic package suitable for both civilian and military trauma in prehospital settings [22]. Our previous studies have further shown that the hemostatic properties of CFDP are equivalent to those of its initial plasma [23,24], supporting a clinical trial to evaluate the safety and efficacy of the FDP in bleeding patients.

Most studies on dried plasma focus on its hemostatic characteristics in comparison with its initial frozen plasma [25,26], efficacy, feasibility, and safety for trauma transfusion [21]. Specifically, global coagulation (e.g., activated partial thromboplastin time) and the activities of specific clotting factors and inhibitors (e.g., fibrinogen, factors V and VIII, and antithrombin) were determined by commercially available test kits and coagulation analyzers to assess the quality of FDP and its stability during storage [20,27]. In addition to being widely applied for hemostatic analysis of whole blood, rotational thromboelastometry (ROTEM) has been used to evaluate the quality and hemostasis of fresh plasma, frozen and thawed plasma, and FDP [23,28]. Although FDP can be stored between 2 and 25 °C for up to 2 years [29], its stability after reconstitution is less studied [30]. According to the current guideline, reconstituted LyoPlas should be used immediately or at the latest within 6 h; however, more detailed information is not available. There is a need to investigate the stability of different FDP products after reconstitution and exposure to extreme temperatures for their optimal use in various environments, with ambient temperatures ranging from well below 0 °C in Arctic areas to above 40 °C in tropical and desert areas. Therefore, we assessed the hemostatic stabilities of CFDP and LyoPlas, respectively, after reconstitution in sterile water under different storage temperatures and static or rocking conditions.

## 2. Materials and Methods

### 2.1. Canadian Freeze-Dried Plasma Product (CFDP)

**Figure 1:**
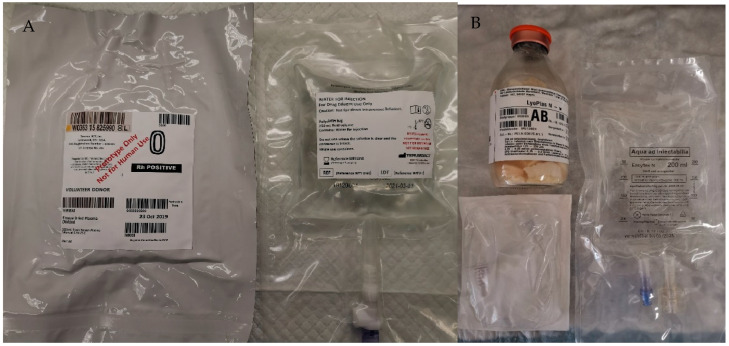
Prototype kit of CFDP (**A**) and packaged unit of LyoPlas (**B**).

CFDP was produced from Canadian-sourced plasma by Canadian Blood Services (CBS) as previously described [23]. Briefly, plasma from Canadian donors was isolated from whole blood units collected in citrate phosphate dextrose anticoagulant that is red blood cell-reduced by centrifugation, leukocyte-reduced (residual counts < 5 × 10^6^ per unit), and frozen at −80 °C. Frozen plasma (FP) units were thawed and pooled to produce plasma pools of 10 units. Each FP pool was then lyophilized using a high-quality 15-shelf freeze-dryer (Lyovapor L-200, BUCHI Corporation, New Castle, DL, USA). One prototype kit consists of 250 mL of CFDP in a plastic bag and another plastic bag containing 250 mL of sterile water for injection (Figure 1A). The two bags can be connected directly to transfer the water. After reconstitution, CFDP units were aliquoted and frozen at −80 °C immediately (designated as baseline 0 h), after storage at 4 °C for 24 h, or at room temperature (RT) for 4 h prior to further analysis.

### 2.2. German Freeze-Dried Plasma Product (LyoPlas)

LyoPlas was kindly provided by Canadian Forces Health Services. The German Red Cross produced LyoPlas from single-donor plasma. One packaged unit includes one glass bottle with 200 mL of FDP, one plastic bag containing 200 mL of water for injection, and one transfer set for transferring the water (Figure 1B). LyoPlas was reconstituted according to the manufacturer’s preparation instructions for transfusion. The reconstituted LyoPlas was stored in 50 mL Falcon tubes at various temperatures (−20 °C, 4 °C, 22 °C, and 35 °C) for up to 1344 h (8 weeks). For 4 °C and above, samples were taken at 2 h, 4 h, 8 h, 24 h, 48 h, 72 h, 96 h, 120 h, 144 h, and 168 h and stored at −80 °C for future analysis. For −20 °C, aliquots were obtained from the reconstituted LyoPlas in the original bottle and stored in Eppendorf tubes for 168 h, 336 h, 672 h, and 1344 h, respectively, to avoid repeated freezing and thawing. In addition, to study the effect of mechanical agitation from soldier presence patrol and transportation, reconstituted LyoPlas was rotated on a rocker (VWR International, Mississauga, ON, Canada) at 18 rpm under 22 °C (22° C R). Sterile techniques were used to handle the reconstitution, sampling, and storage to avoid bacterial contamination.

Global hemostatic tests and specific factor assays were conducted using the ROTEM and Stago Max Compact analyzers, respectively, using standard reagents and procedures recommended by each manufacturer as described below. ROTEM provides a complete evaluation of the process of clot initiation, formation, and fibrinolysis by quantitatively measuring the elasticity of whole blood or plasma as it clots under low shear stress [31,32]. The Stago analyzer enables assays of each specific coagulation factor and inhibitor in plasma, including FDP [27].

### 2.3. Rotational Thromboelastometry (ROTEM)

ROTEM tests (INTEM, EXTEM) were conducted on the reconstituted plasma samples at 37 °C with a ROTEM delta machine using standard reagents and procedures as recommended by the manufacturer (Instrumentation Laboratory, Bedford, MA, USA). The following parameters were recorded for each test: clotting time CT, clot formation time CFT, alpha angle, maximum clot firmness MCF, and clot lysis index LI30. Specifically, CT and MCF were used as two key parameters to assess the quality of freeze-dried plasma (onset of clot formation and maximum clot strength). The ROTEM technology and clinical applications have been well described in the literature [32].

### 2.4. Special Coagulation Testing Hemostatic Assays

Prothrombin time (PT), partial thromboplastin time (PTT), and specific coagulation and fibrinolysis factor assays (fibrinogen, factor V, VIII, antithrombin, D-Dimer, plasminogen, protein C, and protein S) were conducted using Stago Compact Max (Diagnostica Stago, Inc., Parsippany, NJ, USA) following the manufacturer’s instructions.

### 2.5. Statistical Analysis

Percentage changes were calculated as the difference in parameter values between the baseline (0 h) sample and stored samples for different times divided by the baseline to determine the hemostatic stability. Data points are expressed as the mean ± standard deviation. One-sample T tests were used to determine any changes relative to baseline. One-way analysis of variance (ANOVA) with Bonferroni post hoc tests was used to determine any differences in hemostatic properties between the samples stored under different conditions. All statistical analyses were conducted using SPSS Statistics 28 (IBM Corporation, Armonk, NY, USA). A *p* value of less than 0.05 was considered significant.

## 3. Results

We performed global hemostatic tests and specific factor assays to evaluate the short-term and long-term hemostatic stability of CFDP and LyoPlas after reconstitution, respectively.

### 3.1. Short-Term Stability of Reconstituted CFDP

**Figure 2:**
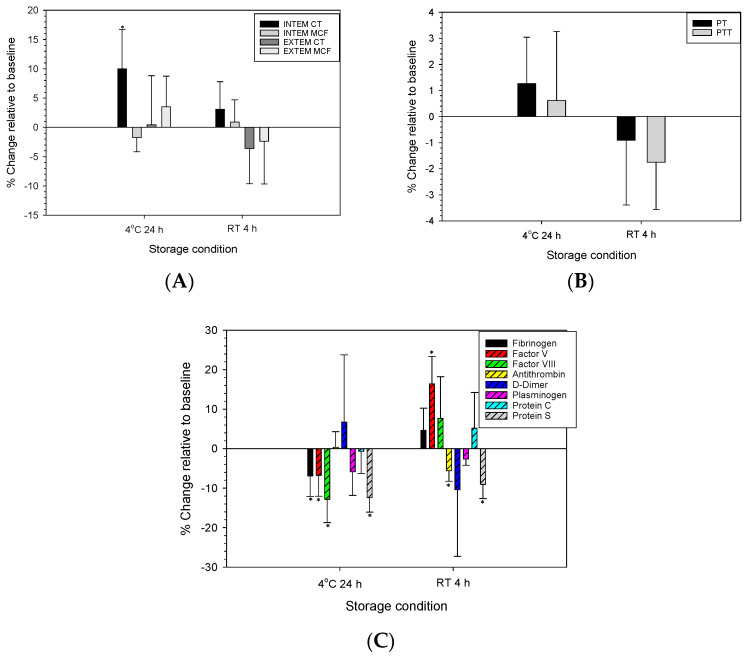
ROTEM (**A**) and Stago tests (**B**,**C**) of reconstituted CFDP stored at 4 °C for 24 h and room temperature (RT) for 4 h, respectively. Data represent mean ± SD (*n* = 3–5). * Significant difference from the baseline (0 h).

As depicted in Figure 2A, compared to initial reconstituted CFDP (baseline 0 h), there were no changes in INTEM MCF, EXTEM CT, and MCF after storage at 4 °C for 24 h and RT for 4 h, respectively, while an increase in INTEM CT close to 10% was seen after storage at 4 °C for 24 h.

Figure 2B,C show no significant changes in Stago measurements for PT, PTT, D-dimer concentration, plasminogen, and protein C activities after storage at 4 °C for 24 h and RT for 4 h, respectively, while decreases in fibrinogen concentration, factors V and VIII, and protein S activities were observed after storage at 4 °C for 24 h, and an increase in factor V activity and decreases in antithrombin and protein S activities relative to the baseline were seen after storage at RT for 4 h.

### 3.2. Long-Term Stability of Reconstituted LyoPlas

**Figure 3:**
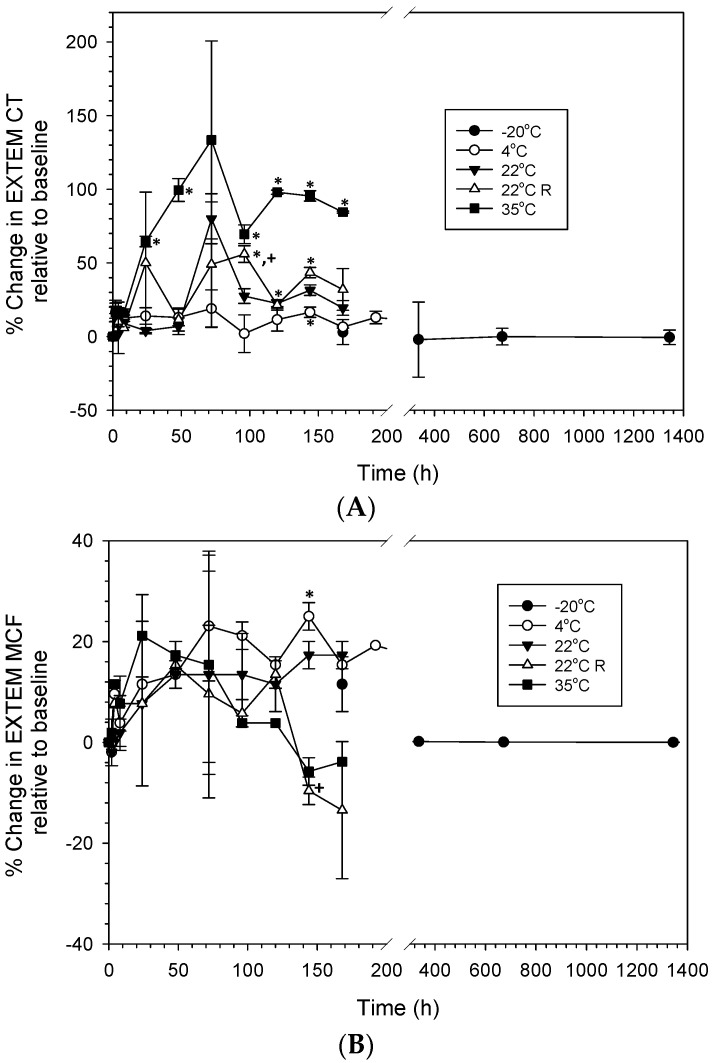
Percent changes in EXTEM CT (**A**) and MCF (**B**) relative to baseline (0 h) of reconstituted LyoPlas stored at different conditions over time. Data represent mean ± SD (*n* = 3). * Significant difference from the baseline (0 h). + Significant difference from the change at the same time under 22 °C.

As expected, different changes in the global hemostatic profiles over time were observed depending on the storage temperature (Figure 3). Profound increases in EXTEM CT were seen at 35 °C after 24-h storage, in contrast with relatively few changes at the lower temperatures, where EXTEM CT was increased after storage at −4 °C for 144 h, at 22 °C for 120 h, and at 22 °C R for 96 h, respectively (Figure 3A). There were no significant changes in EXTEM MCF of reconstituted LyoPlas stored at different conditions over time, except for an increase after storage at −4 °C for 144 h (Figure 3B). No changes in EXTEM CT and MCF were observed when reconstituted LyoPlas was stored at −20 °C, even for 1344 h (8 weeks). Moreover, a comparison of the hemostatic properties between static and rocking conditions at 22 °C demonstrated a larger increase in CT at 96 h and a decrease in MCF at 144 h after storage under rocking, implying a rocking effect on the hemostatic stability.

**Figure 4:**
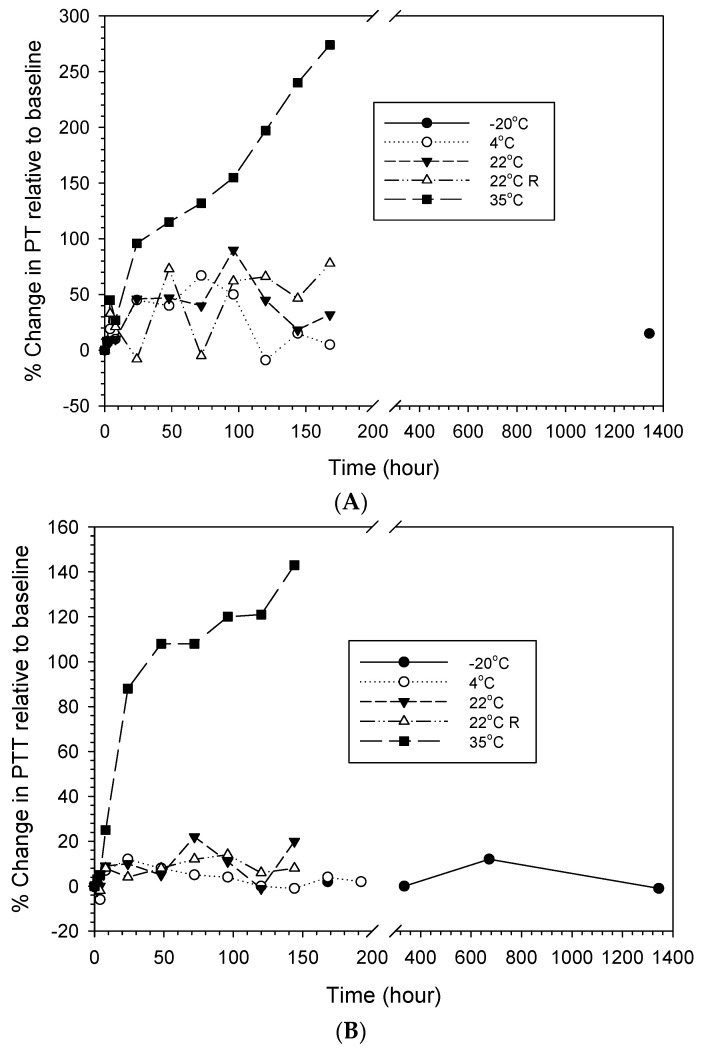
Percentage changes in PT (**A**) and PTT (**B**) relative to baseline (0 h) of reconstituted LyoPlas stored at different conditions over time.

Figure 4 shows the changes in PT and PTT after reconstitution of LyoPlas and storage at each condition over time. Although no statistical analysis could be performed with a single measurement, more progressive and rapid increases in PT and PTT were seen after storage at 35 °C for 8 h compared to those at 22 °C and below, where large variations (up to 78% in PT and 22% in PTT) with no clear trends were observed.

**Figure 5:**
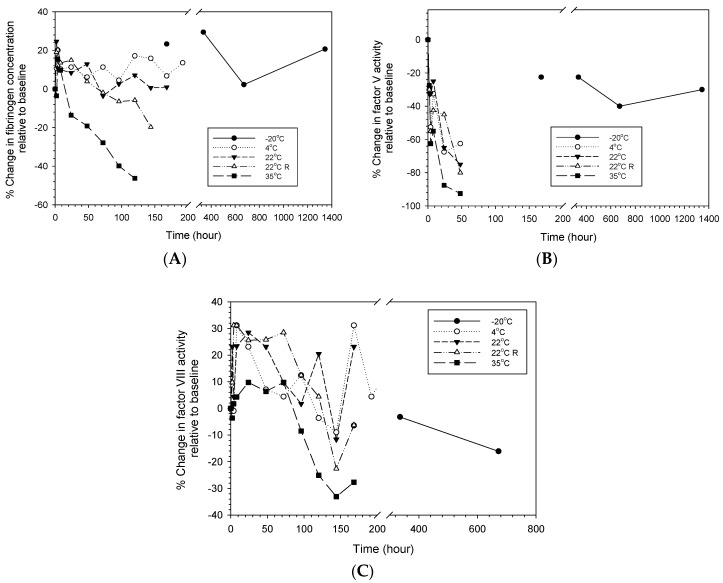
Percentage changes in coagulation factors of reconstituted LyoPlas stored at different conditions. (**A**) fibrinogen; (**B**) factor V; (**C**) factor VIII.

Figure 5 depicts the changes in coagulation factors of reconstituted LyoPlas stored at different conditions over time. Similar to PT and PTT, decreased stability in specific hemostatic profiles with increasing storage temperatures was observed, particularly at 35 °C. In addition, there were different changes in the coagulation factor profiles over time, depending on the storage temperature. Specifically, fibrinogen and factor VIII underwent the most progressive decreases in their concentration and activity at 35 °C, beginning at 8 and 72 h, respectively, compared to the changes at 22 °C and below, while factor V showed dramatic decreases in its activity by more than 60% after 48-h storage under all storage conditions except −20 °C, at which an approximate −20% decrease was observed.

**Figure 6:**
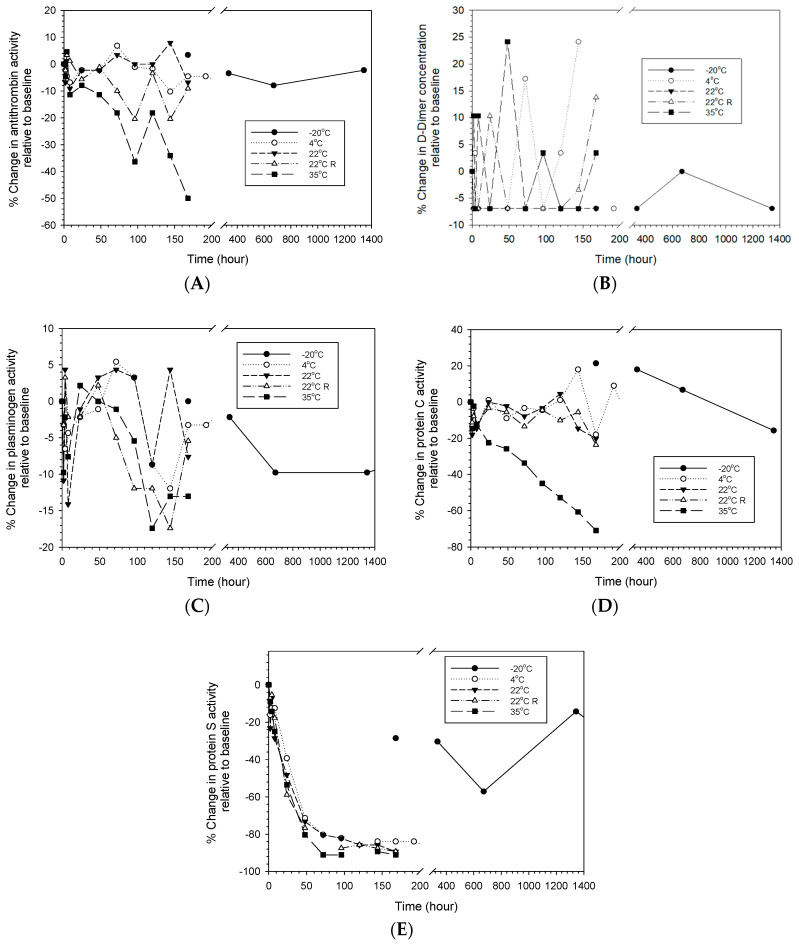
Percentage changes in anticoagulation factors of reconstituted LyoPlas stored at different conditions. (**A**) antithrombin; (**B**) D-dimer; (**C**) plasminogen; (**D**) protein C; (**E**) protein S.

Figure 6 shows different changes in the fibrinolysis mediator activities of reconstituted LyoPlas stored at different temperatures over time. Similar to the coagulation factors, antithrombin and protein C lost their activities more progressively at 35 °C than at 22 °C and below, while protein S dramatically lost its activity from the start of the experiment and up to 80% after 48-hour storage under all storage conditions except at −20 °C, at which decreases of about 20% or more were observed. D-dimer and plasminogen showed variations between −20% and 25%, and there was no clear effect of the storage condition as storage time increased.

## 4. Discussion

Compared to traditional prehospital resuscitation fluids, such as crystalloids, FDP can not only serve as a volume expander but also retain all the benefits of its source plasma, being physiologically relevant, thereby preventing hemodilution and acidosis, possessing hemostatic properties, and correcting the endotheliopathy of trauma while providing superior logistical advantages [10]. Therefore, in the absence of low-titer whole blood, FDP may serve as a great alternative for prehospital hemorrhage management, especially without access to tranexamic acid and in austere environments.

Previous studies on FDP products indicated increases in PT, PTT, and decreases in the levels of specific coagulation factors and inhibitors (fibrinogen, factors V, FVIII, and FXI, antithrombin, plasminogen, protein C, protein S, and von Willebrand factor) over time when stored in a dry state under different temperature-controlled and uncontrolled field conditions, but most global and specific hemostatic functions were within clinical ranges when stored at 4 °C and RT for up to 2 years [20,22,33,34,35].

In addition to the stability of FDP in a dry state, it is important to examine the stability of FDP after reconstitution for optimal use in prehospital settings, which can be influenced by field conditions, in particular storage temperature and time. Our results confirmed that the hemostatic properties of reconstituted FDP as measured by ROTEM were not altered at 4 °C or RT for a short duration, although long-term stability over a range of storage conditions was reduced and worth further investigation.

The key finding of this study is the short-term hemostatic stability of reconstituted CFDP at 4 °C or RT and temperature-dependent changes of LyoPlas after reconstitution and storage for a long duration, as measured by global ROTEM and plasma factor-specific Stago tests. The hemostatic properties of reconstituted CFDP after storage at 4 °C or RT for a short duration are consistent with the shelf life of reconstituted LyoPlas, as suggested by its stability studies after storage at 4 °C over 6 days [30] and RT over 48 h [20].

Although the global hemostatic profiles as measured by ROTEM indicated minimal short-term changes after storage at 4 °C and RT, some specific factors in the coagulation cascade were compromised, including factors V and VIII, which are most susceptible to loss of their hemostatic activities over the drying process [36] and storage time compared to other plasma proteins [37].

In addition, evaluation of the hemostatic stability of reconstituted CFDP and LyoPlas showed that global coagulation functions and key coagulation and fibrinolysis factors were affected by storage temperature differently, beginning to change at different storage times.

Overall, our study indicated that factor V and protein S were most liable, and D-dimer and plasminogen were most stable after reconstitution and storage over the range of temperature and time investigated. In contrast, different stabilities were reported for the clotting factor and inhibitor activities in the literature. One study showed the largest decrease in the activity of factor VIII by 24.3%, followed by factors XI, IX, V, and fibrinogen over 6 days after reconstitution and storage of LyoPlas at 4 °C, while storage at 4 °C for 6 h led to a significant decrease in factor VIII activity by 14.9%, slight decreases in factor V and antithrombin activities by 1.3%, slight increases in protein C activity and free protein S, and no change in fibrinogen concentration [30]. Another study on storage of reconstituted LyoPlas at RT showed most decreases in factor VIII and protein S activities, less than 10% in the first 6 h, and 66% and 50% of starting activities within 48 h, while factor V lost its activity approximately from 87% to 80%, and fibrinogen and antithrombin remained stable within 48 h [20]. In contrast, French FDP (FLYP) showed a larger decline of 38% from the baseline value in factor V activity, followed by a decline of 17% in factor VIII activity and 10% in fibrinogen concentration, but less than 10% changes in PT and PTT after reconstitution and storage at 4 °C for 24 h [29]. The discrepancies could be due to differences in measurement protocols, including instruments and reagents used for each test, storage temperature and time, type of FDP-producing plasma (single-donor, pooled, or solvent-/detergent-treated plasma), and donor variabilities. For example, the lower stability of protein S in our study of reconstituted CFDP and LyoPlas could be due to the difference in the assays to detect protein S (activity versus antigen methods). Factors V and VIII were more stable, while protein S was less stable in solvent/detergent-treated plasma [38,39] and FDP [40], respectively.

Given its representation for clotting factor and inhibitor activities, changes in ROTEM CT imply overall global hemostatic functions. Despite the statistically significant decrease in some of the clotting factors (fibrinogen, factors V, VIII) and inhibitors (antithrombin, protein S), ROTEM CT values of CFDP remained within normal limits, suggesting sufficient clot formation at least after storage at 4 °C for 24 h and RT for 4 h, respectively. On the other hand, ROTEM CT was also mostly affected at 35 °C compared to that at the lower temperatures in the long-term stability study, which is consistent with the temperature-dependent changes in plasma clotting factors. In contrast, ROTEM MCF was less impaired by the decrease in fibrinogen concentration, despite their association [41].

As mechanical agitation associated with soldier patrols and transportation on rugged terrain in a combat environment may cause denaturation of proteins [42], we studied the reconstituted LyoPlas stability under the mechanical agitation by rocking the sample at 18 rpm. More impairment on ROTEM CT and MCF was only seen at one time point compared to the static condition at the same temperature of 22 °C. The rocking effect needs to be further evaluated by Stago measurements with a large sample size.

The stability of coagulation factors is dependent on a combination of several factors, such as interactions with other proteins (factor VIII is stabilized by the Von Willebrand factor), temperature sensitivity (factors V and VIII are easily degraded at higher temperatures), and enzymatic degradation in plasma (coagulation proteases are prone to cleavage) [43,44]. As a result, certain coagulation factors, including factors V and VIII, are more susceptible to loss of function.

The clinical significance of the changes in each clotting factor is unclear. Fibrinogen and factor V are clotting factors often depleted in trauma patients with coagulopathy [45,46]. Their stability in hemostatic functions could be most important for reconstituted FDP for trauma resuscitation. On the other hand, traumatic coagulopathy may be influenced by several interactions between plasma proteins and the surrounding tissues, such as the endothelium, without the clear dominance of a single factor [47]. Alternatively, factor VIII is a sensitive factor routinely used as the quality marker of plasma, as quality control demands factor VIII activity above 70% for the freshly collected plasma unit [48].

A limitation of the study is the small sample size due to the shortage of FDP units available for analysis, especially for the long-term stability of reconstituted LyoPlas. To complete the current evaluation, further research is needed to ensure no potentially harmful degradation product is created and to investigate any bacterial contamination and growth during storage, especially at RT and above. In addition, the long-term hemostatic stability and effects of agitation of CFDP were not comprehensively assessed due to its limited availability for analysis since it is still under development. Consequently, we opted to comprehensively assess the long-term hemostatic stability and response to agitation of LyoPlas instead. With that said, we anticipate that our findings are applicable to other FDP products, which should demonstrate a similar hemostatic profile under these long-term conditions. These findings would provide a basis for improved inventory management and better support for the use of FDP for severe trauma resuscitation in various field conditions.

## 5. Conclusions

We have confirmed the short-term stability of CFDP in global hemostatic properties after reconstitution and storage at RT, consistent with the shelf life of reconstituted LyoPlas. The long-term stability analyses of reconstituted LyoPlas suggest that temperature-dependent changes in global hemostatic functions and specific factor activities vary. The post-reconstitution hemostatic stability of FDP products would decrease over time with increasing storage temperatures, with a significant loss of hemostatic properties at 35 °C compared to 22 °C or below. Therefore, based on our findings presented here, we recommend: (1) the shelf-life of FDP should be based on storage temperature; (2) FDP should not be stored above RT; and (3) FDP should be administered immediately or within 4 h after reconstitution to maintain peak hemostatic function.

## Data Availability

Data are contained within the article.
